# The Degree of *Helicobacter pylori* Infection Affects the State of Macrophage Polarization through Crosstalk between ROS and HIF-1*α*

**DOI:** 10.1155/2020/5281795

**Published:** 2020-12-08

**Authors:** Ying Lu, Jianfang Rong, Yongkang Lai, Li Tao, Xiaogang Yuan, Xu Shu

**Affiliations:** ^1^Department of Gastroenterology, The First Affiliated Hospital of Nanchang University, No. 17 Yongwaizheng Street, Nanchang, 330006 Jiangxi Province, China; ^2^Department of Gastroenterology, The Second Affiliated Hospital of Nanchang University, No. 1 Minde Road, Nanchang, 330006 Jiangxi Province, China; ^3^Department of Gastroenterology, Longgang District People's Hospital of Shenzhen, Shenzhen, China

## Abstract

**Methods:**

The expression of CD86, CD206, and HIF-1*α* in the gastric mucosa was evaluated through immunohistochemistry. RAW 264.7 cells were cocultured with *H. pylori* at various multiplicities of infection (MOIs), and iNOS, CD86, Arg-1, CD206, and HIF-1*α* expression was detected by Western blot, PCR, and ELISA analyses. ROS expression was detected with the fluorescent probe DCFH-DA. Macrophages were also treated with the ROS inhibitor NAC or HIF-1*α* inhibitor YC-1.

**Results:**

Immunohistochemical staining revealed that the macrophage polarization state was associated with the progression of gastric lesions and state of *H. pylori* infection. The MOI of *H. pylori* affected macrophage polarization, and *H. pylori* enhanced the expression of ROS and HIF-1*α* in macrophages. A low MOI of *H. pylori* promoted both the M1 and M2 phenotypes, while a high MOI suppressed the M2 phenotype. Furthermore, ROS inhibition attenuated HIF-1*α* expression and switched macrophage polarization from M1 to M2. However, HIF-1*α* inhibition suppressed ROS expression and inhibited both the M1 phenotype and the M2 phenotype. Inhibition of ROS or HIF-1*α* also suppressed the activation of the Akt/mTOR pathway, which was implicated in *H. pylori*-induced macrophage polarization.

**Conclusions:**

Macrophage polarization is associated with the progression of gastric lesions and state of *H. pylori* infection. The MOI of *H. pylori* influences the macrophage polarization state. Crosstalk between ROS and HIF-1*α* regulates *H. pylori*-induced macrophage polarization via the Akt/mTOR pathway.

## 1. Introduction

Macrophages play a central role in host defense and the inflammatory response and are significant components of the body's innate and adaptive immune systems [1, 2]. Macrophage polarization is a process by which macrophages respond to microenvironmental signals and stimuli in specific tissues and acquire specific phenotypes. Polarized macrophages can be functionally divided into two major categories: classically activated macrophages (M1) and alternatively activated macrophages (M2) [3]. M1 macrophages are characterized by the secretion of tumor necrosis factor-*α* (TNF-*α*), inducible nitric oxide synthase (iNOS), interleukin- (IL-) 6, IL-1 beta (IL-*β*), and other chemokines and play essential roles in the inflammatory response, antitumor response, and promotion of host immunity [4, 5]. M2 macrophages can secrete a great number of anti-inflammatory cytokines: transforming growth factor-*β* (TGF-*β*), IL-10, CD206, and arginase-I (Arg-1) [6]. It is generally believed that M1 macrophages perform proinflammatory, bactericidal, and cancer-suppressing functions, while M2 macrophages participate in parasite containment, tumor progression, and tissue remodeling promotion and have immunomodulatory functions [3]. Macrophages maintain a dynamic balance between the M1 and M2 phenotypes in healthy subjects. Once macrophages are extremely skewed toward either the M1 phenotype or the M2 phenotype over time, they can cause the progression of many diseases, such as rheumatoid arthritis, obesity, and cancer [7].

*Helicobacter pylori* (*H. pylori*), a gram-negative microaerophilic bacterium, infects approximately half of the population worldwide [8]. Long-term infection with *H. pylori* can result in chronic gastritis, peptic ulcers, and gastric adenocarcinoma [9]. *H. pylori* recruits macrophages to the gastric mucosa and induces them to secrete proinflammatory cytokines and chemokines, causing inflammation and damage to the gastric mucosa [10–12]. Several studies have reported the influence of *H. pylori* infection on macrophage polarization. *H. pylori* not only protects against chronic colitis by promoting M2 polarization [13] but also enhances M1 polarization in human and mouse gastric macrophages, leading to the occurrence of *H. pylori-*associated atrophic gastritis [14]. However, there have been no studies further investigating the role of the multiplicity of infection (MOI) of *H. pylori* in macrophage polarization, as illustrated in our study, although a previous study reported that high and low MOIs of *H. pylori* played converse roles in B lymphocyte apoptosis [15].

Reactive oxygen species (ROS), which are mainly derived from superoxide anions (O_2_^−^), hydrogen peroxide (H_2_O_2_), and hydroxyl radicals (OH^−^), are cooperative or independent regulators of cellular signaling in response to different environmental stimuli rather than simply a harmful byproduct of cell metabolism [16]. It has been reported that ROS are involved in DNA repair, cell cycling, cell differentiation, chromatin remodeling, self-renewal, and other cell processes [17]. Furthermore, ROS play an essential role in the regulation of macrophage polarization. A reduced ROS level suppresses the M1 phenotype and promotes macrophage polarization into the M2 phenotype [18–21]. However, the link between ROS and *H. pylori*-induced macrophage polarization has not been well clarified. Hypoxia-inducible factor 1 *α* (HIF-1*α*) is involved in cell proliferation, tumor angiogenesis, apoptosis [22], infection [23], inflammatory diseases [24], and innate immune responses. Under normoxia, HIF-1*α* undergoes rapid degradation, but it has been reported that HIF-1*α* expression can be enhanced and maintained by either endogenous or *H. pylori*-induced elevated ROS level in the gastric mucosa under normoxia [25]. Matak et al. demonstrated that HIF-1*α* contributed to M1 macrophage polarization in *H. pylori*-mediated gastritis; interestingly, HIF-1*α* also had an anti-inflammatory function at the same time [26], which indicated that HIF-1*α* may play dual roles in macrophage polarization. Nevertheless, the role of HIF-1*α* in macrophage polarization, especially in the context of *H. pylori*-associated polarization, has not been well explored.

*H. pylori* infection, ROS, and HIF-1*α* are involved in the process of regulating macrophage polarization. However, the role and specific mechanisms of *H. pylori* in macrophage polarization, especially the effect of the MOI of *H. pylori*, remain poorly understood. The roles of ROS and HIF-1*α* in *H. pylori*-induced macrophage polarization remain to be further explored. Hence, this study, for the first time, investigated the MOI of *H. pylori*, the interaction between ROS and HIF-1*α*, and their roles in regulating *H. pylori*-induced macrophage polarization. In addition, we explored the effects of ROS and HIF-1*α* on the Akt/mTOR signaling pathway in this process, as studies have reported a central role for the Akt/mTOR pathway in macrophage polarization [27].

## 2. Materials and Methods

### 2.1. Cell Culture

RAW 264.7 cells from mice were cultured in Dulbecco's modified Eagle's medium (HyClone, Logan, UT, USA) containing 10% fetal bovine serum (Gibco, CA, USA) and 1% penicillin/streptomycin (Solarbio Biotechnology, Beijing, China) at 37°C in a 5% CO_2_ atmosphere. After digestion, cells were inoculated into six-well plates and cocultured with the *H. pylori* standard strain 43504 when the cells reached a logarithmic growth phase. The *H. pylori* strain 43504 was grown on solid brucella agar plates supplemented with 5% fresh sheep blood and 1% penicillin/streptomycin at 37°C under microaerophilic conditions. After coculture with *H. pylori* at different MOIs for 9 h, cells were collected for RT-PCR, Western blot, and ELISA analyses. Cells were also treated with the ROS inhibitor N-acetylcysteine (NAC; 10 mM, Sigma-Aldrich, MO, USA) or HIF-1*α* inhibitor [3-(5′-hydroxymethyl-2′-furyl)-1-benzylindazole] (YC-1; 10 *μ*M, Sigma). Macrophages were further incubated with the Akt inhibitor LY294002 (20 *μ*mol/L) (Selleck, USA) or the mTOR inhibitor rapamycin (20 nmol/L) (Selleck).

### 2.2. Western Blotting

After washing twice with ice-cold phosphate-buffered saline (PBS), total protein was extracted from treated cells with a mixture of RIPA lysis buffer (Beyotime Biotechnology, Shanghai, China), benzenesulfonyl fluoride (PMSF) (Beyotime Biotechnology), and a protein phosphatase inhibitor (Sigma). The protein concentration was determined via the BCA method after centrifugation at 12,000 rpm for 10 min. Protein was separated on a 10% SDS polyacrylamide gel, transferred to nitrocellulose membranes, and blocked in a blocking solution at room temperature for 1 h. The primary antibodies used were as follows: anti-HIF-1*α* (#36169), anti-Akt (#2920), anti-p-Akt (Ser473) (#4060), anti-mTOR (#2983), anti-p-mTOR (Ser2448) (#5536), and anti-*β*-actin (#4970S). All the primary antibodies were obtained from Cell Signaling Technology (Beverly, MA, USA). After incubation with primary antibodies at 4°C overnight, the membranes were washed three times with Tris-buffered saline-Tween (TBST) (Solarbio Biotechnology) and then incubated with a horseradish peroxidase-conjugated secondary antibody (Zhongshan Golden Bridge Biotech; dilution 1 : 5,000) for 1 h at room temperature. Protein bands were visualized with an enhanced chemiluminescence kit (Thermo Fisher Scientific, Suwanee, GA, USA). The band intensity of target proteins was normalized to that of *β*-actin.

### 2.3. Real-Time Quantitative PCR Analysis

Total RNA was extracted from RAW 264.7 cells utilizing the RNA simple Total RNA Kit (TIANGEN Biotech, Beijing, China) and converted to cDNA with the Fast Quant RT Kit (TIANGEN Biotech) according to the manufacturer's instructions. NanoDrop 2000 (Thermo Fisher Scientific) was used to measure the concentration and purity of the isolated RNA. Real-time quantitative PCR was conducted to detect the transcriptional levels of CD86, CD206, Arg-1, iNOS, and HIF-1*α* by using the Step-One™ Real-Time PCR System (Applied Biosystems, CA, USA). The transcriptional levels of target genes were normalized to that of GAPDH.

### 2.4. ELISA

The levels of iNOS and Arg-1, markers related to macrophage polarization, were measured with the Mouse iNOS ELISA Kit (Elabscience Biotechnology Co., Ltd., Wuhan, China) and Mouse Arg-1 ELISA Kit (Elabscience Biotechnology Co.), respectively, according to the manufacturer's instructions.

### 2.5. Gastric Specimens and Immunohistochemistry

We collected 240 endoscopic biopsy specimens from the Yudu County People's Hospital of Ganzhou and the Digestive Endoscopy Center of the First Affiliated Hospital of Nanchang University and obtained patient consent before collecting the specimens. We also recorded relevant patient information, which showed that there were no significant differences in the age and sex distributions among the patients (Supplemental Table S1). These specimens included equal numbers (60 cases) of chronic nonatrophic gastritis (CNAG), intestinal metaplasia (IM), dysplasia (Dys), and gastric cancer (GC) samples. Each group contained *H. pylori* negative (*H. pylori* (-), 20 cases), *H. pylori* moderately positive (*H. pylori* (+), 20 cases), and *H. pylori* strongly positive (*H. pylori* (+++), 20 cases), with the quantities of *H. pylori* based on a previous study [28]. Four slices of each subject were used to analyze the expression of target proteins by immunohistochemical staining, and the results were evaluated by two pathologists who were blinded to the identity of the samples and scored for intensity (level 0-3) and frequency (level 0-4) (a total of 100 cells were counted in five random fields). For statistical analysis, using the formula intensity × frequency, the levels of each target protein were reported according to an expression score with a range of 0 to 12.

### 2.6. ROS Detection

The level of ROS in RAW 264.7 cells was examined with Molecular Probes™ CM-H2DCFDA (Thermo Fisher Scientific). Cells were incubated with a CM-H2DCFDA staining solution at 37°C in the dark for 30 min and washed 3 times with sterile PBS. Then, 200 *μ*L of cell suspension was added to the 24-well plate and imaged using a high-content fluorescence microscope.

### 2.7. Statistical Analysis

All statistical analyses were conducted with SPSS 20.0. The results included in this study were obtained from at least three independent experiments and are represented as the mean ± SEM. The statistical significance of variables was analyzed by ANOVA. For multiple comparisons, post hoc analyses were evaluated with LSD correction. A *p* value < 0.05 was considered statistically significant.

## 3. Results

### 3.1. The State of Macrophage Polarization Was Associated with Progression from CNAG to GC and Correlated with the State and Quantity of *H. pylori* Infection

**Figure 1:**
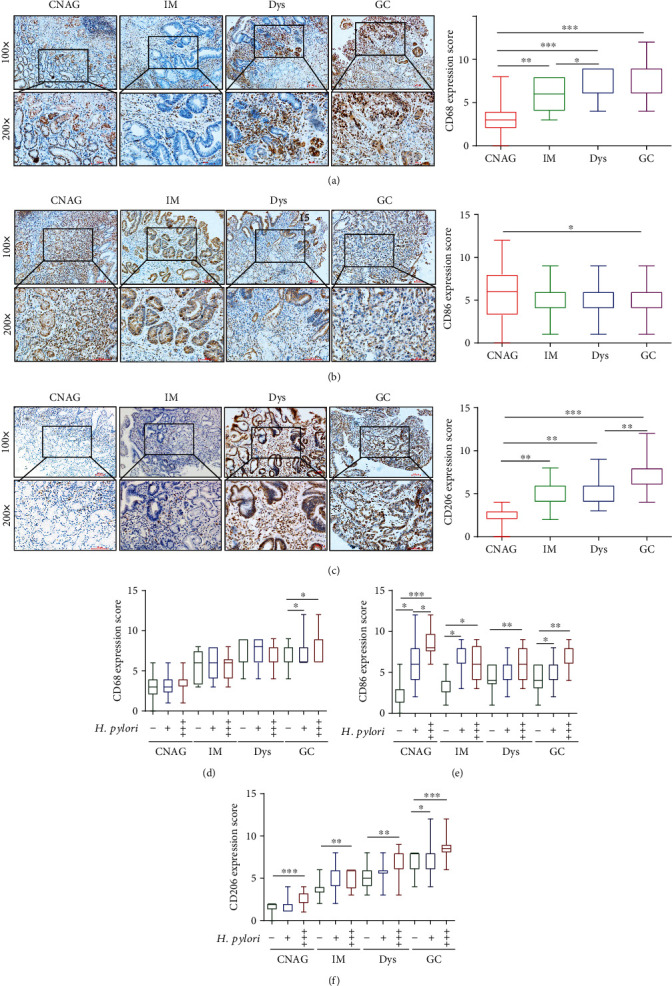
The state of macrophage polarization was associated with the progression from chronic nonatrophic gastritis to gastric cancer and correlated with the state of *H. pylori* infection. Representative images of immunohistochemical staining for CD68 (a), CD86 (b), and CD206 (c) in human gastric mucosa samples with CNAG, IM, Dys, or GC (magnification 200x, scale bars = 50 *μ*m). Scores were assessed, and statistical comparisons were conducted to evaluate the results for the expression of CD68, CD86, and CD206, as shown on the right side of the representative images. Levels of CD68 (d), CD86 (e), and CD206 (f) compared between *H. pylori* (-), *H. pylori* (+), and *H. pylori* (+++) gastric tissue samples at different stages. ^∗^*p* < 0.05, ^∗∗^*p* < 0.01, and ^∗∗∗^*p* < 0.001.

Studies have reported that macrophage polarization is associated with gastritis [14] and GC [29]. To further explore the correlation between macrophage polarization and clinical progression from CNAG to GC, we detected the expression of macrophage polarization markers, including CD86 (indicative of M1 polarization) and CD206 (indicative of M2 polarization), and the macrophage marker CD68 in 240 human gastric tissue samples diagnosed with CNAG, IM, Dys, or GC through immunohistochemistry. As shown in Figures 1(a)–1(c), with the progression of gastric lesions, the expression of CD68 and CD206 increased gradually, whereas the level of CD86 was higher in CNAG but lower in IM, Dys, and GC, which indicated that there was macrophage infiltration in gastric lesions and the macrophage polarization state was implicated in the progression of gastric lesions. We found that macrophages appeared to exhibit the M1 phenotype in the early stage of gastric lesions, such as CNAG, while they tended to display the M2 phenotype in advanced pathological stages, such as GC. To determine the role of *H. pylori* in gastric macrophage polarization, we further divided these 240 human gastric tissue samples with different stages of pathological changes into three groups: *H. pylori* (-), *H. pylori* (+), and *H. pylori* (+++). Interestingly, as suggested in Figures 1(d)–1(f), the expression of CD68, CD86, and CD206 was correlated with the *H. pylori* infection status in gastric tissues. We discovered that in all stages of gastric lesions, the levels of CD206 and CD86 in the *H. pylori*-positive groups were higher than those in the *H. pylori*-negative group. CD68 expression in gastric carcinomas was higher in the *H. pylori*-positive groups than in the *H. pylori*-negative group. Additionally, the expression levels of CD86 and CD206 in the *H. pylori* (+) and *H. pylori* (+++) groups were different, which indicated that the quantity of *H. pylori* might affect macrophage polarization. Overall, we discovered that the state of macrophage polarization was implicated in the progression of gastric lesions and associated with the state and quantity of *H. pylori* infection.

### 3.2. HIF-1*α* Expression Was Positively Related to Markers of Macrophage Polarization in Gastric Tissues and Associated with the State and Quantity of *H. pylori* Infection

**Figure 2:**
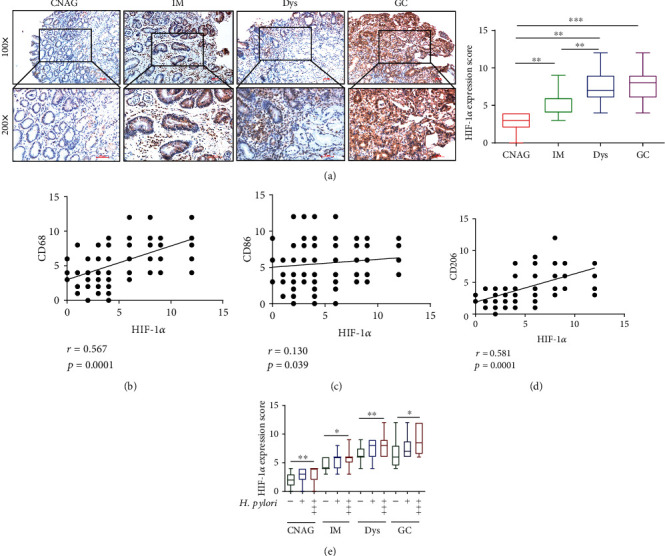
HIF-1*α* expression was positively related to markers of macrophage polarization in gastric tissues and associated with the state and quantities of *H. pylori* infection. Representative images of immunohistochemical staining for HIF-1*α* (a) in human gastric mucosa samples with CNAG, IM, Dys, or GC (magnification 200x, scale bars = 50 *μ*m). Scores were assessed, and statistical comparisons were conducted to evaluate the results for the expression of HIF-1*α*. Pearson correlation analyses showing that the expression score of HIF-1*α* was positively associated with the scores of CD68 (b), CD86 (c), and CD206 (d). HIF-1*α* expression (e) between *H. pylori* (-), *H. pylori* (+), and *H. pylori* (+++) gastric tissue samples at different stages. ^∗^*p* < 0.05, ^∗∗^*p* < 0.01, and ^∗∗∗^*p* < 0.001.

As HIF-1*α* is related to M2 macrophage polarization in cancer [30], we speculated whether HIF-1*α* can influence the progression of gastric lesions by regulating gastric macrophage polarization. Thus, we tested the expression of HIF-1*α* during the progression from CNAG to GC. As shown in Figure 2(a), HIF-1*α* expression increased gradually with the progression of gastric lesions. We further investigated the relationship between HIF-*α* and gastric macrophage polarization and found that HIF-1*α* levels were positively associated with the expression of CD68, CD86, and CD206 via Pearson correlation analysis (Figures 2(b)–2(d)). Additionally, the level of HIF-1*α* was correlated with the state and quantity of *H. pylori* (Figure 2(e)). All these results suggested that HIF-1*α* was correlated with gastric macrophage polarization and associated with the state and quantity of *H. pylori* infection during the progression of gastric lesions.

### 3.3. The MOI of *H. pylori* Affected the State of Macrophage Polarization and Expression of HIF-1*α* and ROS

**Figure 3:**
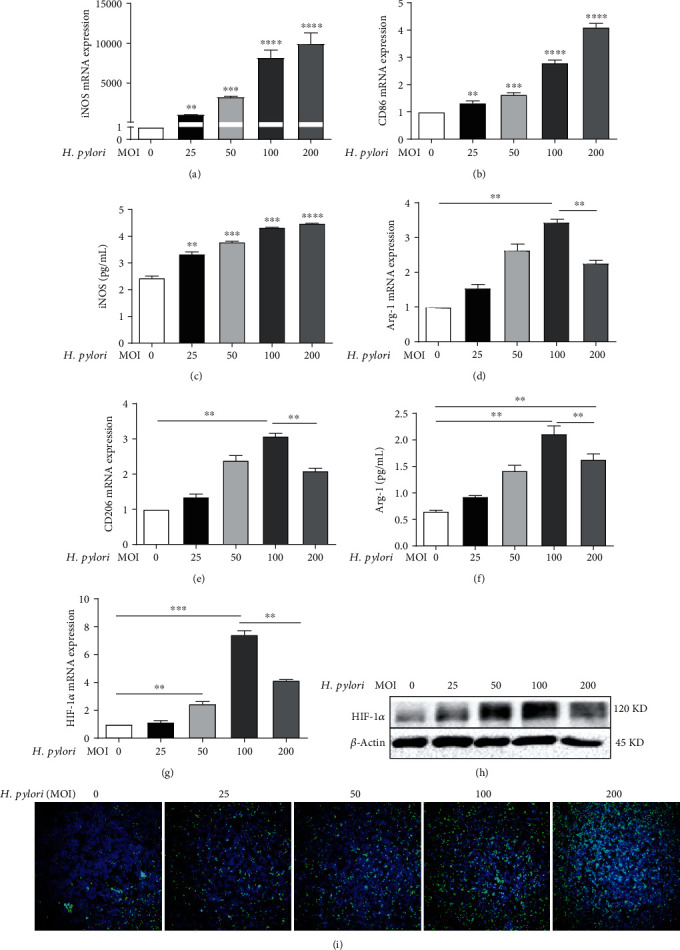
The MOI of *H. pylori* affected the state of macrophage polarization and expression of HIF-1*α* and ROS. Macrophages (RAW 264.7 cells) were incubated with *H. pylori* at various MOIs for 9 h. The mRNA expression levels of iNOS (a), CD86 (b), Arg-1 (d), and CD206 (e) measured by real-time PCR. ELISA detection of iNOS (c) and Arg-1 (f) levels. HIF-1*α* expression analyzed by real-time PCR (g) and immunoblotting (h). ROS expression evaluated with the fluorescent probe DCFH-DA (i), green: ROS, blue: nucleus. All experiments were independently repeated three times. ^∗^*p* < 0.05, ^∗∗^*p* < 0.01, and ^∗∗∗^*p* < 0.001.

Our previous study revealed that the ROS level was elevated by *H. pylori* infection in an MOI-dependent manner in GC [31], and in the present study, we found that the state and quantity of *H. pylori* infection affected macrophage polarization and HIF-1*α* expression. We speculated whether the MOI of *H. pylori* can influence the state of macrophage polarization and the expression of HIF-1*α* and ROS in macrophages; thus, we conducted the following experiments. We cocultured the macrophage cell line RAW 264.7 with *H. pylori* strain 43504 at various MOIs (0, 25, 50, 100, and 200) for 9 h, and the expression of iNOS, CD86, CD206, Arg-1, HIF-1*α*, and ROS was detected. As shown in Figures 3(a)–3(c), the levels of CD86 and iNOS were found to be much higher in the *H. pylori* infection groups than in the uninfected control group (*p* < 0.05). Moreover, the levels of CD86 and iNOS were positively associated with the MOI of *H. pylori*, which suggested that *H. pylori* could promote macrophage polarization toward the M1 phenotype in an MOI-dependent manner. Likewise, the levels of CD206 and Arg-1 were higher in the *H. pylori*-infected groups than in the uninfected control group. However, interestingly, although the increases in the CD206 and Arg-1 levels were dependent on the MOI at low MOIs (25, 50, and 100), the expression of CD206 and Arg-1 was suppressed slightly in the MOI 200 group (the MOI 200 group compared to the MOI 100 group, *p* < 0.01) (Figures 3(d)–3(f)), which suggested that M2 macrophage polarization was inhibited by a high MOI of *H. pylori* compared with a low MOI. Similarly, the mRNA and protein expression of HIF-1*α* showed trends similar to those of CD206 and Arg-1 (Figures 3(g) and 3(h)). As expected, the level of ROS in macrophages was also significantly increased by *H. pylori* in an MOI-dependent manner (Figure 3(i)). All these results indicated that the MOI of *H. pylori* influenced the macrophage polarization status and expression of HIF-1*α* and ROS in macrophages.

### 3.4. ROS and HIF-1*α* Influenced the Macrophage Polarization Induced by *H. pylori*

**Figure 4:**
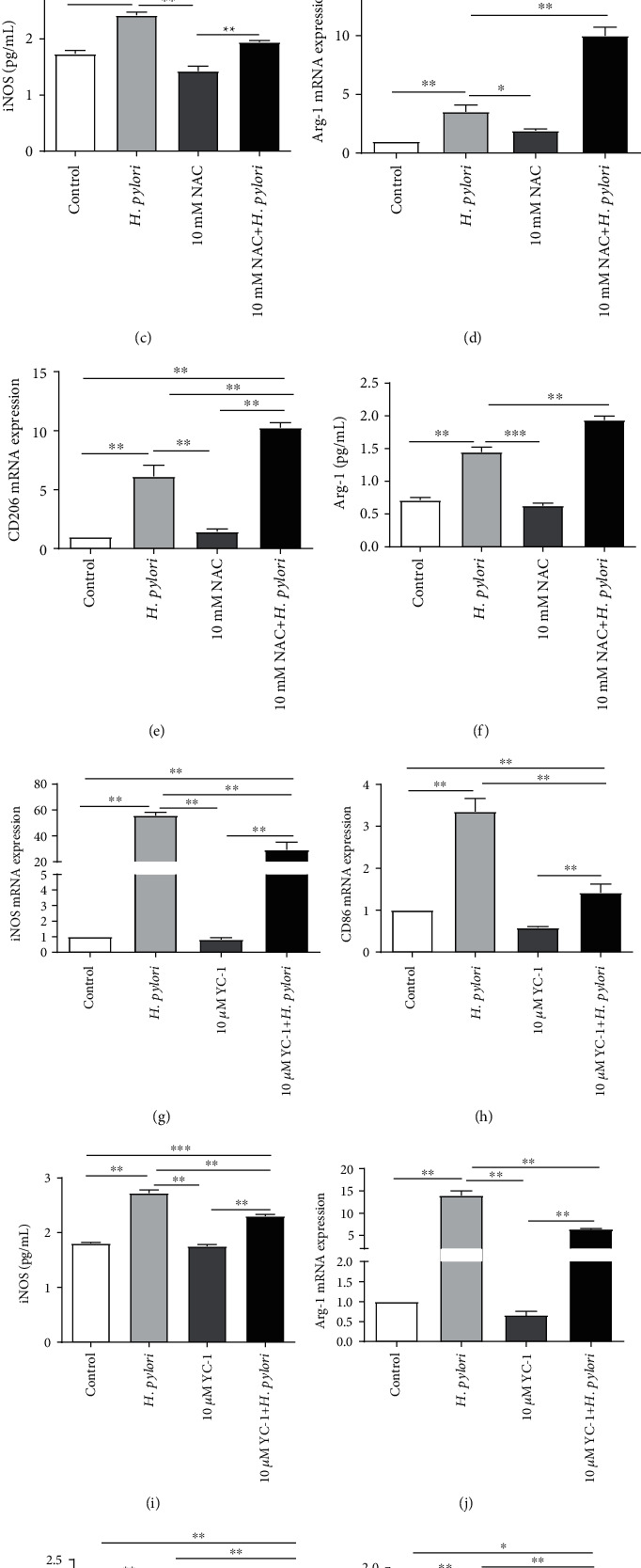
ROS and HIF-1*α* influenced the macrophage polarization induced by *H. pylori*. (a–f) RAW 264.7 cells were incubated with *H. pylori* (MOI = 100) alone or in combination with NAC (10 mM). NAC treatment inhibited the M1 phenotype but promoted the M2 phenotype. (g–l) RAW 264.7 cells were incubated with *H. pylori* (MOI = 100) alone or in combination with YC-1 (10 *μ*M). YC-1 treatment inhibited both M1 and M2 macrophage polarization. The expression of iNOS, CD86, CD206, and Arg-1 was detected by real-time PCR ((a–d), (g), (h), (j), and (k)). The expression of iNOS (c, i) and Arg-1 (f, l) was also analyzed by ELISA. All experiments were independently repeated three times. ^∗^*p* < 0.05, ^∗∗^*p* < 0.01, and ^∗∗∗^*p* < 0.001.

Given that *H. pylori* infection affected macrophage polarization and upregulated the levels of ROS and HIF-1*α* in macrophages, we further evaluated whether ROS and HIF-1*α* expression influence the macrophage polarization induced by *H. pylori*. We treated RAW 264.7 cells with the ROS inhibitor NAC (10 mM), and decreases in iNOS and CD86 mRNA expression and increases in Arg-1 and CD206 mRNA levels were observed in the cells treated with the combination of NAC and *H. pylori* compared to the cells treated with *H. pylori* alone by using RT-PCR (Figures 4(a), 4(b), 4(d), and 4(e)). We also detected iNOS and Arg-1 levels by ELISA and further determined the upregulation of Arg-1 expression and downregulation of iNOS expression in groups treated with the combination of NAC and *H. pylori* compared with group*s* treated with *H. pylori* alone (Figures 4(c) and 4(f)). These results demonstrated that ROS inhibition suppressed macrophage polarization toward the M1 phenotype and promoted macrophage polarization toward the M2 phenotype. In contrast, we discovered that the HIF-1*α* inhibitor YC-1 (10 *μ*M) decreased the elevated expression of iNOS, CD86, Arg-1, and CD206 induced by *H. pylori* utilizing RT-PCR (Figures 4(g), 4(h), 4(j), and 4(k)) and ELISA (Figures 4(i) and 4(l)), which indicated that inhibition of HIF-1*α* restrained M1 and M2 macrophages. In summary, our results indicated that ROS and HIF-1*α* could regulate *H. pylori*-mediated macrophage polarization.

### 3.5. Crosstalk between ROS and HIF-1*α* in *H. pylori*-Infected Macrophages

**Figure 5:**
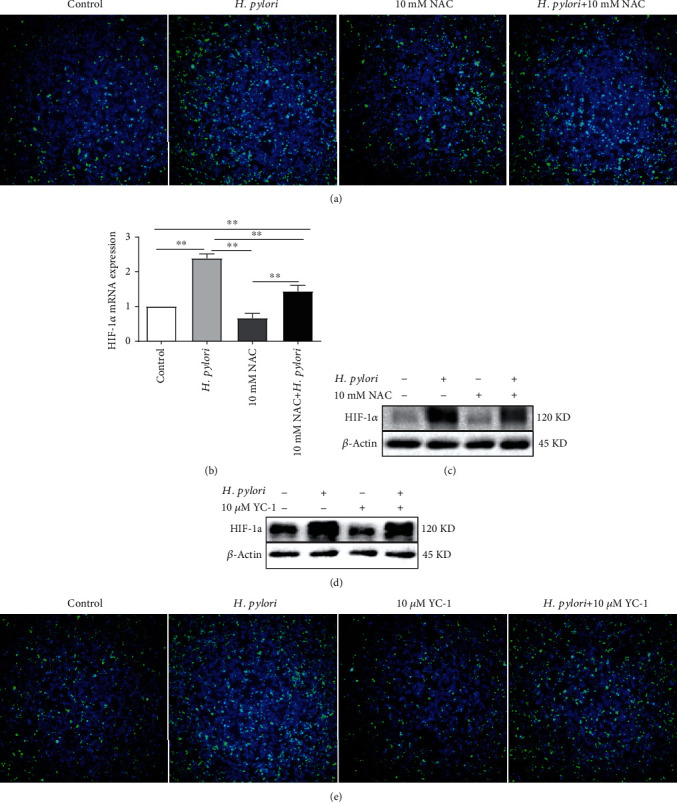
The crosstalk between ROS and HIF-1*α* in *H. pylori*-infected macrophages. (a–c) RAW 264.7 cells were incubated with *H. pylori* (MOI = 100) alone or in combination with NAC (10 mM). ROS inhibition decreased the enhanced expression of HIF-1*α* induced by *H. pylori*. (d, e) RAW 264.7 cells were incubated with *H. pylori* (MOI = 100) alone or in combination with YC-1 (10 *μ*M). HIF-1*α* inhibition downregulated the augmented expression of ROS induced by *H. pylori*. ROS expression was detected with the fluorescent probe DCFH-DA (green: ROS, blue: nucleus). HIF-1*α* expression was tested by real-time PCR and Western blotting. Representative images are shown. All experiments were independently repeated three times. ^∗^*p* < 0.05, ^∗∗^*p* < 0.01, and ^∗∗∗^*p* < 0.001.

Based on the results above and our previous research showing that ROS inhibition decreased the *H. pylori*-induced enhancement of HIF-*α* expression in BALB/c mice (Supplemental Figures 1(a)-1(c)), we suspected whether there was an interaction between ROS and HIF-1*α* in macrophages during *H. pylori* infection. Thus, we treated RAW 264.7 cells with 10 mM NAC (a ROS inhibitor) alone or in combination with the *H. pylori* strain. The data shown in Figure 5(a) suggested that NAC treatment attenuated both the ROS expression and the augmented ROS level induced by *H. pylori* infection. Moreover, the mRNA and protein levels of HIF-1*α* were suppressed in cells treated with NAC alone, and the *H. pylori*-induced elevations in HIF-1*α* mRNA and protein expression levels were also sharply decreased by NAC treatment (Figures 5(b) and 5(c)), which indicated that ROS inhibition could inhibit HIF-1*α* expression in macrophages. Interestingly, when we treated RAW 264.7 cells with 10 *μ*M YC-1 (a HIF-1*α* inhibitor), similar results were observed. YC-1 attenuated the expression of HIF-1*α* and ROS compared with the control group. Furthermore, YC-1 obviously decreased the augmented *H. pylori*-induced levels of HIF-1*α* (Figure 5(d)) and ROS (Figure 5(e)) in *H. pylori-*infected RAW 264.7 cells. Taken together, these results revealed that there was crosstalk between ROS and HIF-1*α* in *H. pylori*-infected macrophages.

### 3.6. ROS and HIF-1*α* Regulated *H. pylori*-Induced Macrophage Polarization via the Akt/mTOR Pathway

**Figure 6:**
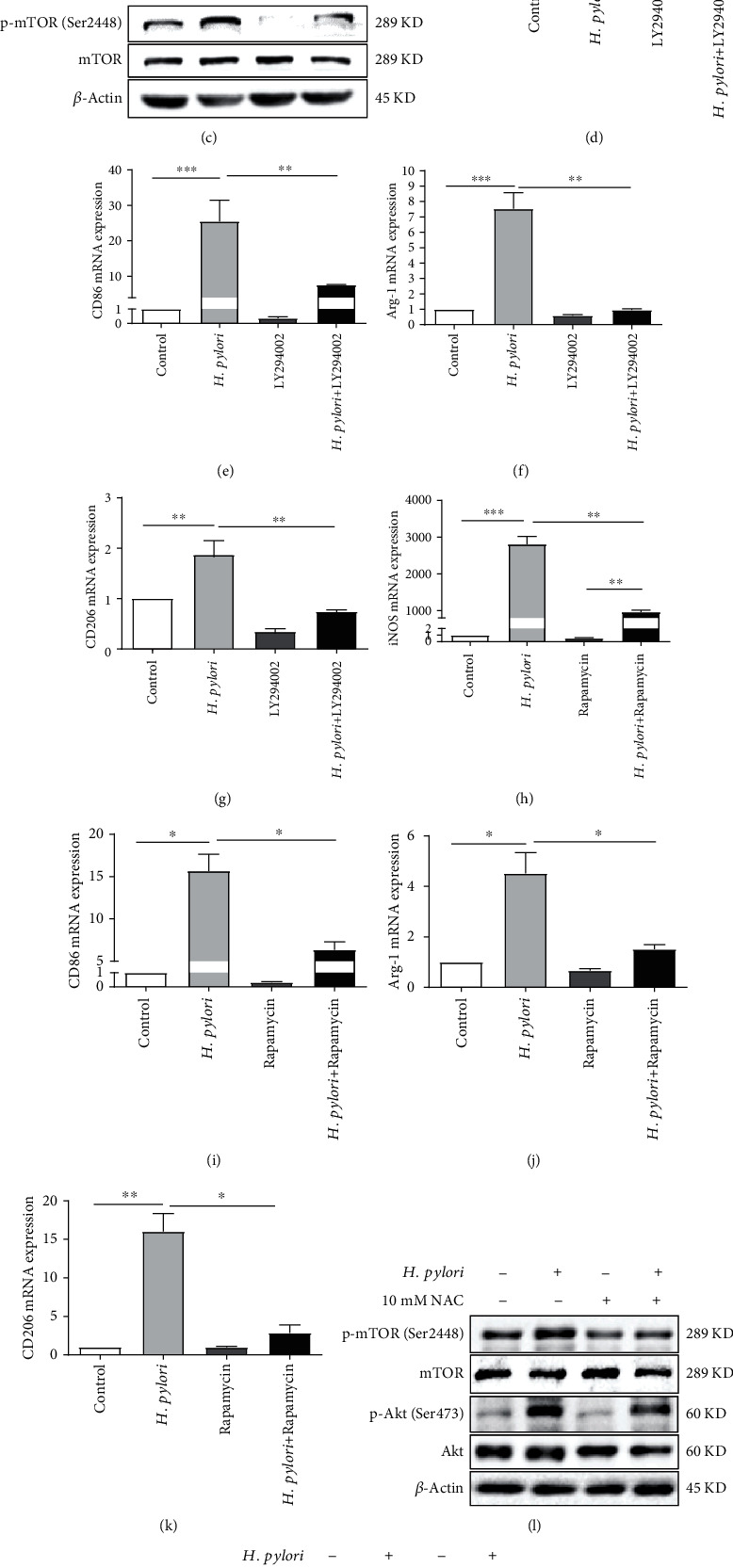
ROS and HIF-1*α* regulated *H. pylori*-induced macrophage polarization via the Akt/mTOR pathway. Increased expression of p-mTOR (Ser2448) and p-Akt (Ser473) was observed in RAW 264.7 cells treated with *H. pylori* at different MOIs for 9 h (a). RAW 264.7 cells were treated with *H. pylori* (MOI = 100), LY294002 (20 *μ*mol/L), rapamycin (20 nmol/L), the combination of *H. pylori* and LY294002 (20 *μ*mol/L), or the combination of *H. pylori* and rapamycin (20 nmol/L). LY294002 and rapamycin significantly attenuated the levels of p-Akt (Ser473) (b) and p-mTOR (Ser2448) (c), as well as the M1 (d–g) and M2 (h–k) phenotypes induced by *H. pylori.* RAW 264.7 cells were incubated with *H. pylori* (MOI = 100) alone or in combination with NAC (10 mM) (l) or YC-1 (10 *μ*M) (m). Both NAC and YC-1 treatment reduced the augmented p-mTOR (Ser2448) and p-Akt (Ser473) levels induced by *H. pylori*. All experiments were independently repeated three times.

Several studies have demonstrated a crucial role for the Akt/mTOR pathway in the M1 and M2 polarization of macrophages [32–34]. However, there have been no studies on the function of the Akt/mTOR pathway in *H. pylori*-induced macrophage polarization. As shown in Figure 6(a), the protein levels of p-Akt (Ser473) and p-mTOR (Ser2448) were much higher in *H. pylori*-infected macrophages than in uninfected controls, while the total protein expression of Akt and mTOR was not different between the *H. pylori*-infected and control groups. Moreover, as the MOI of *H. pylori* increased, the levels of p-Akt (Ser473) and p-mTOR (Ser2448) showed trends toward increasing gradually at low MOIs and decreasing slightly at high MOIs (MOI = 200), which was similar to the results observed for HIF-1*α*, Arg-1, and CD206. We further treated cells with an inhibitor of Akt (LY294002, 20 *μ*mol/L) or mTOR (rapamycin, 20 nmol/L) and observed sharp attenuation of the enhanced expression of p-Akt (Ser473) or p-mTOR (Ser2448) induced by *H. pylori* infection (Figures 6(b) and 6(c)). Moreover, as shown in (Figures 6(d)–6(k)), the augmented levels of iNOS, CD86, Arg-1, and CD206 in *H. pylori*-infected macrophages were reduced by LY294002 and rapamycin, which suggested that the Akt/mTOR pathway plays an important role in *H. pylori-*mediated macrophage polarization. Since we found that the crosstalk between ROS and HIF-1*α* regulated the polarization of macrophages induced by *H. pylori* infection, we further explored the roles of ROS and HIF-1*α* in this pathway. Western blot analysis showed that both NAC (10 mM) and YC-1 (10 *μ*M) extremely inhibited the levels of p-Akt (Ser473) and p-mTOR (Ser2448) in macrophages and attenuated the elevated p-Akt (Ser473) and p-mTOR (Ser2448) expression induced by *H. pylori* infection (Figures 6(l) and 6(m)). All these data indicated that ROS and HIF-1*α* might regulate *H. pylori*-induced macrophage polarization via the Akt/mTOR pathway.

## 4. Discussion

In this study, we found that *H. pylori* infection was related to macrophage polarization, as reported in several previous studies [13, 14]. Moreover, clinical specimens revealed that different quantities of *H. pylori* infection had different effects on macrophage polarization. Thus, we speculated whether the MOI of *H. pylori* is associated with macrophage polarization, as reported in a previous study showing that a low MOI of *H. pylori* suppressed B lymphocyte apoptosis, while a high MOI promoted B lymphocyte apoptosis [15]. The present study is the first to demonstrate that the MOI of *H. pylori* is associated with the state of macrophage polarization. We found that a low MOI of *H. pylori* promoted the M1 and M2 phenotypes of macrophages, while a high MOI partially inhibited the M2 phenotype compared with low MOIs. This indicated that macrophages were in a mixed state of M1 and M2 cells in the context of a low MOI, but with an increased MOI, M1 macrophages were enhanced, while M2 macrophages were suppressed. Whereas in studies of clinical specimens, compared to that in the *H. pylori* (+) group, the expression of M2-related markers in the *H. pylori* (+++) group did not show a decreasing trend. These contradictory results might be explained by the following two factors: one is that the MOI of in vitro experiments does not exactly match the positive grade of *H. pylori* in clinical specimens, and the concentration of *H. pylori* in the *H. pylori* (+++) group may not have reached the level of the high MOI (MOI = 200); the other is the insufficient number of clinical specimens. However, clinical specimens still revealed certain differences between the *H. pylori* (+) and *H. pylori* (+++) groups.

In our study, we found that *H. pylori* infection enhanced the ROS level in an MOI-dependent manner, which was consistent with our previous study [31]. We also discovered that HIF-1*α* expression in macrophages treated with different MOIs of *H. pylori* showed a trend similar to those of markers of the M2 phenotype, which we suspected would be explained by the dual role of ROS. A previous study revealed that there is a positive feedback loop between ROS and HIF-*α*: ROS upregulate and stabilize HIF-1*α* expression; in turn, elevated HIF-1*α* expression can increase the ROS level. However, when the ROS amount increases to a certain level, it may activate some molecules that in turn downregulate the expression of ROS and HIF-1*α* [35]. Thus, we surmised that the extremely high ROS level induced by the high MOI might slightly suppress the expression of HIF-1*α* through potential molecular mechanisms.

As previously reported, ROS play a critical role in macrophage polarization [36, 37], but the link between ROS and *H. pylori*-induced macrophage polarization has not been identified. In this study, we discovered that inhibiting ROS with NAC inhibited M1 polarization and contributed to M2 polarization mediated by *H. pylori* infection and that ROS inhibition downregulated the expression of HIF-1*α*, which was consistent with a previous study in which ROS production induced by *H. pylori* infection led to constant expression and stabilization of HIF-1*α* [25]. For HIF-1*α*, a high HIF-1*α* level is involved in increases in M2 polarization and accelerates hepatocellular carcinoma progression [30]. Moreover, recruitment of M1 macrophages is dependent on the presence of HIF-1*α* [38, 39]. Our study, for the first time, demonstrated that HIF-1*α* contributed to both M1 and M2 polarization induced by *H. pylori* infection. Moreover, *H. pylor*i-mediated M1 and M2 polarization was attenuated by HIF-1*α* inhibition with YC-1, and a reduction in the ROS level was also observed with this treatment. Hence, we concluded that ROS combined with HIF-1*α* promoted M1 polarization and that HIF-1*α* enhanced M2 polarization when cells were treated with a low MOI of *H. pylori*. In contrast, upon treatment with a high MOI of *H. pylori*, M1 polarization was maintained by ROS, while M2 polarization was partially suppressed, which might be due to the decrease in the HIF-*α* level induced by extremely high ROS expression. However, we did not identify the specific molecular mechanism underlying the mutual regulation between ROS and HIF-1*α* in this study, and this mechanism needs to be further elucidated in our future studies. Although many studies have illustrated that ROS and HIF-1*α* are involved in macrophage polarization, we are the first to discover the crosstalk between ROS and HIF-1*α* in *H. pylori*-induced macrophage polarization, and the expression of ROS and HIF-1*α* was associated with the MOI of *H. pylori.*

Activation of the Akt/mTOR pathway has been determined to play a central role in the regulation of M1 and M2 macrophage polarization in various diseases [27, 40–43]. M2 macrophage polarization induces tamoxifen resistance through the activation of the PI3K/Akt/mTOR pathway in breast cancer [44]. In the present study, elevated levels of Akt and mTOR phosphorylation were observed in macrophages treated with *H. pylori*. Inhibition of the Akt/mTOR pathway greatly inhibited the levels of p-Akt and p-mTOR, resulting in reductions in both M1 and M2 macrophage polarization induced by *H. pylori*. More importantly, both inhibition of ROS and inhibition of HIF-1*α* significantly attenuated the elevated levels of p-Akt and p-mTOR induced by *H. pylori* in macrophages. Hence, we concluded that ROS and HIF-1*α* could regulate *H. pylori*-mediated macrophage polarization via the Akt/mTOR pathway.

Long-term sustained *H. pylori* infection can cause chronic gastritis, peptic ulcers, and gastric adenocarcinoma [9]. During the histopathological Correa cascade of gastric tumorigenesis (from CNAG to GC), M2 macrophage numbers increased gradually, while M1 macrophage numbers showed a trend toward a slight decrease, which was consistent with previous studies that reported that M1 macrophages are mainly involved in proinflammatory processes, while M2 macrophages are associated with tumor transformation [45–47]. Tumor-recruited M2 macrophages contribute to GC metastasis [48] and enhance the resistance of gastric cells to cisplatin treatment [49]. Accordingly, the M2 phenotype of macrophages might be involved in the progression of *H. pylori*-associated gastric carcinoma, which needs to be clarified in our future studies.

In conclusion, our study, for the first time, shows that the MOI of *H. pylori* affects the state of macrophage polarization and the expression of HIF-1*α* and ROS in macrophages. Additionally, ROS and HIF-1*α* regulate *H. pylori*-mediated macrophage polarization via the Akt/mTOR pathway, and there is crosstalk between ROS and HIF-1*α* during macrophage polarization induced by *H. pylori*. Our study describes a new mechanism of *H. pylori*-induced macrophage polarization. Further investigations into the correlation among ROS, HIF-1*α*, *H. pylori* infection, and gastric carcinoma could lead to the development of novel strategies for the therapy of *H. pylori*-associated GC.

## Data Availability

The datasets supporting the conclusions of this article are included within the article and supplemental files.

## Supplementary Materials

Supplementary Materials Supplemental materials contain one table, one Excel spreadsheet, and figures which include statistical graphs of the results of multiple Western blot experiments.
