# Supplementary material for: The Degree of Helicobacter pylori Infection Affects the State of Macrophage Polarization through Crosstalk between ROS and HIF-1α
Source: Oxid Med Cell Longev. 2020 Dec 8;2020:5281795. doi: 10.1155/2020/5281795 (PMC7746446; doi:10.1155/2020/5281795)
Supplement: Supplementary Materials — Supplemental materials contain one table, one Excel spreadsheet, and figures which include statistical graphs of the results of multiple Western blot experiments. [file 5281795.f1.docx]

**Table S1:** Gender and age distribution among groups.

| group | case | gender | | Age(year)  (Mean ± SD) |
| --- | --- | --- | --- | --- |
|  |  | female | male |  |
| CNAG | 60 | 39 | 21 | 53.6±8.4 |
| IM | 60 | 34 | 26 | 52.8±11.7 |
| Dys | 60 | 30 | 30 | 54.9±12.3 |
| GC | 60 | 21 | 30 | 59.5±14.1 |
| total | 240 | 133 | 107 | 55.2±11.6 |


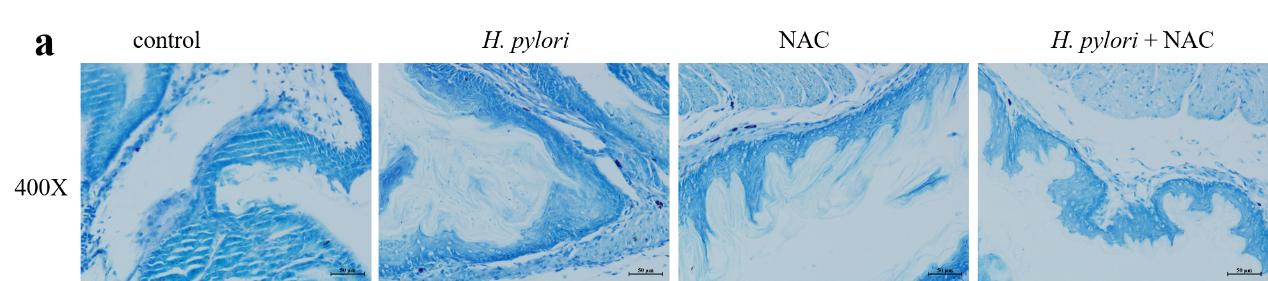


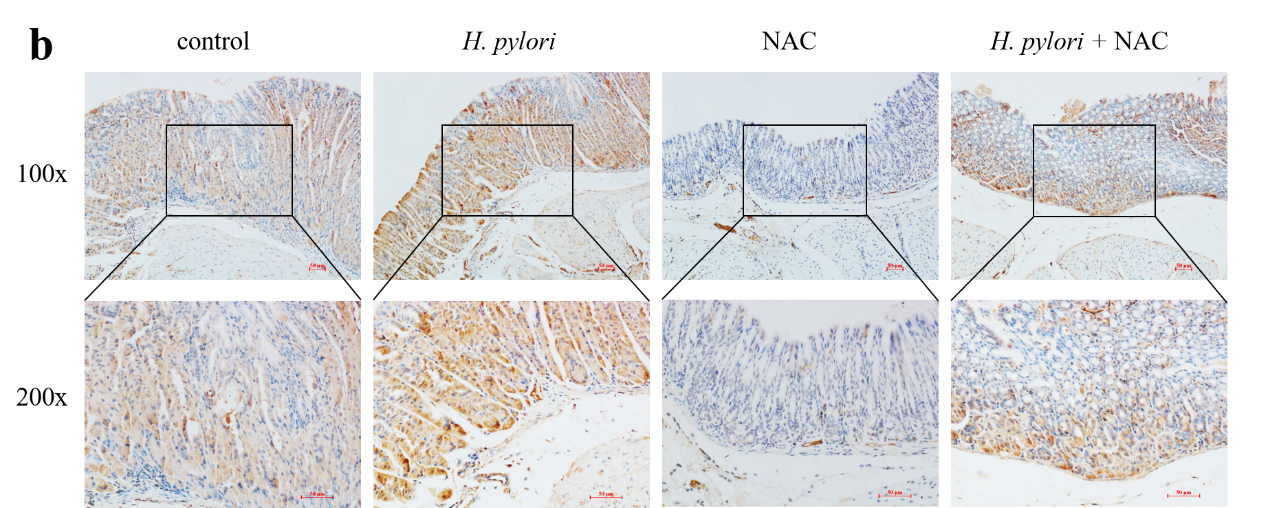


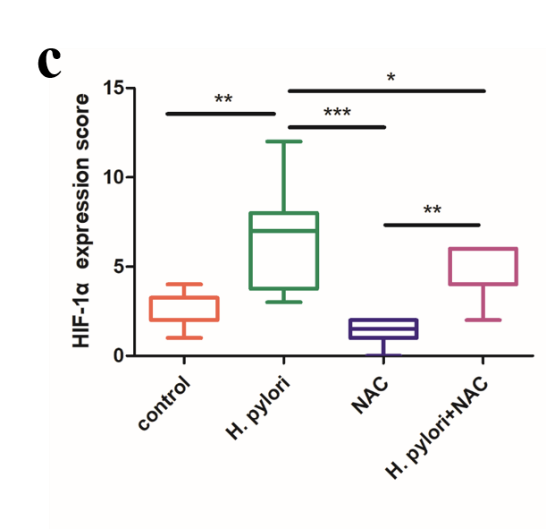


**Figure S1：**ROS influenced the expression of HIF-1α in the gastric mucosa of mice after *H. pylori* infection. (**A**) The gastric mucosa of BALB/c mice after treated with *H. pylori* alone or in combination with NAC for 24w; (**B, C**) ROS inhibition attenuated *H. pylori*-induced augmented HIF-1α expression in gastric mucosa of BALB/c mice.

**Quantitative results of western blots (Please double click the form below)**

**Statistical Graphs for the Results of multiple Western blot Experiments

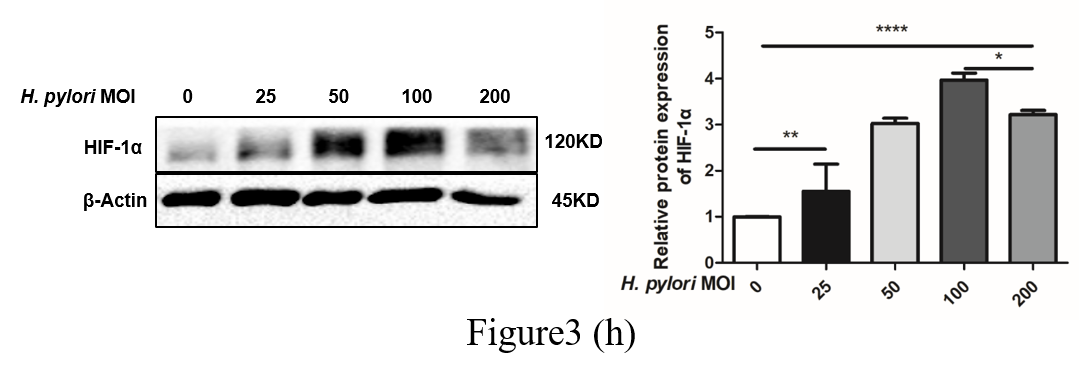
**

**
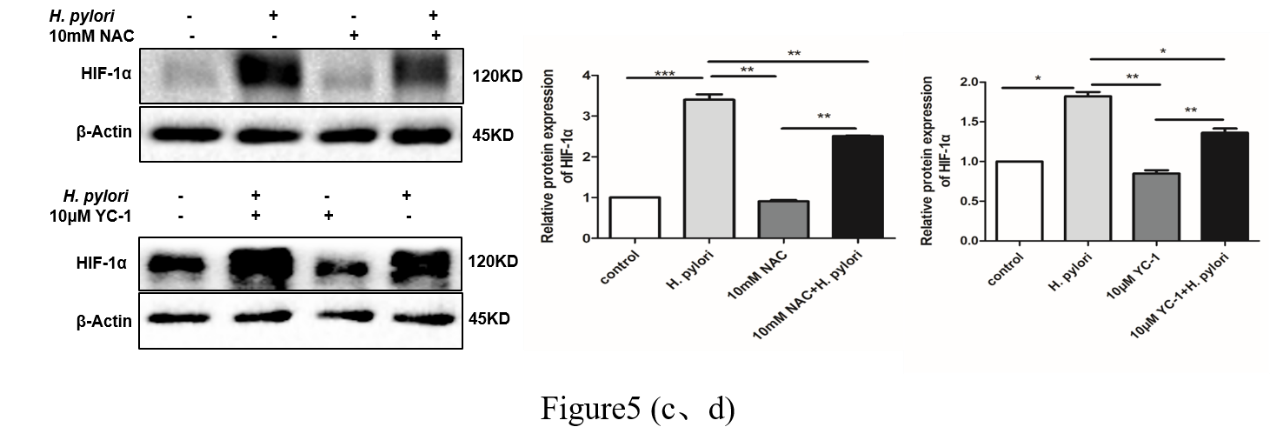
**

**
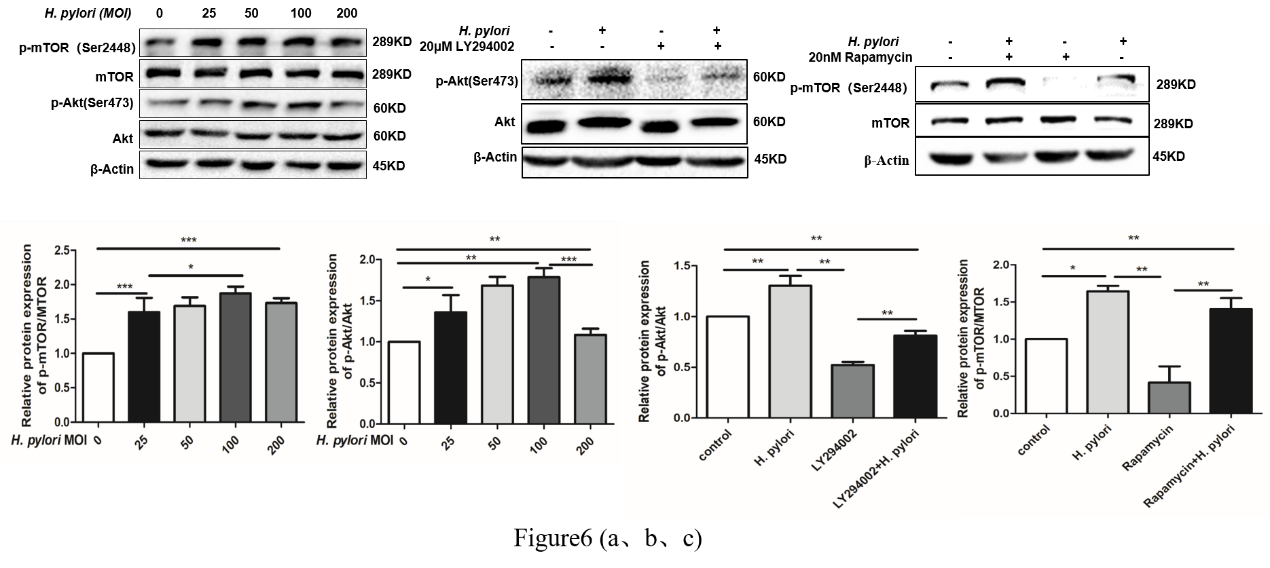
**

**
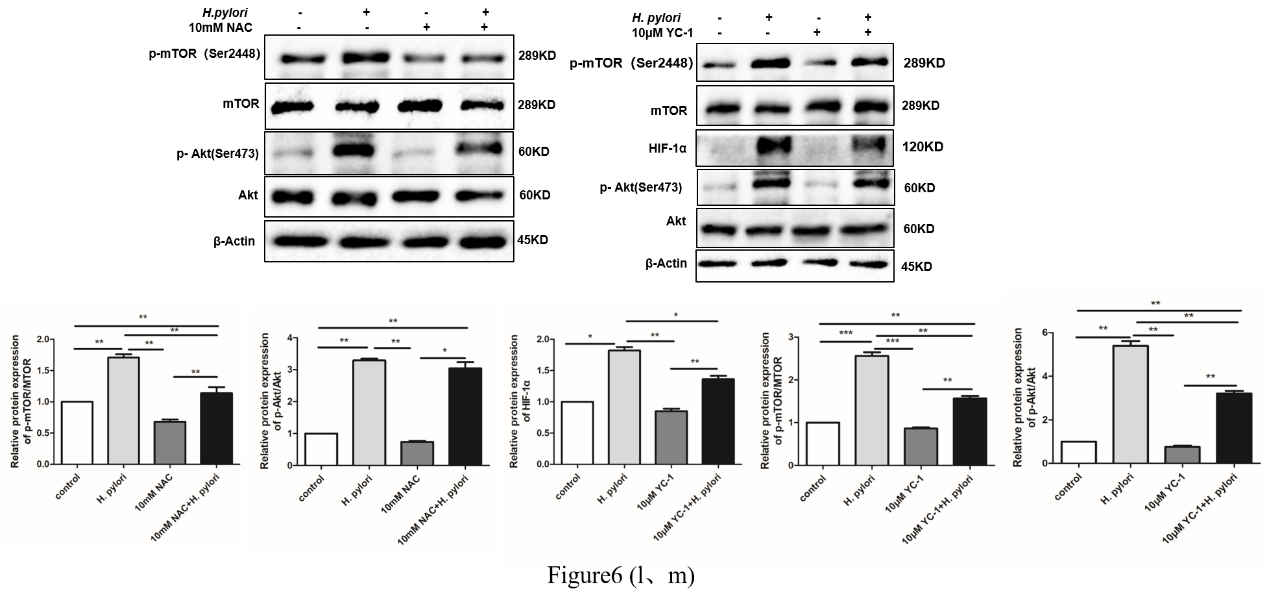
**
